# Dapagliflozin a glucose-regulating drug with diuretic properties in subjects with type 2 diabetes

**DOI:** 10.1111/dom.12127

**Published:** 2013-06-05

**Authors:** H J Lambers Heerspink, D de Zeeuw, L Wie, B Leslie, J List

**Affiliations:** 1 Department of Clinical Pharmacology, University Medical Center Groningen, University of GroningenGroningen, The Netherlands; 2 Global Clinical Research, Bristol-Meyer-SquibbPrinceton, NJ, USA; 3 Global Biometric Sciences, Bristol-Meyer-SquibbHopewell, NJ, USA

**Keywords:** blood pressure, dapagliflozin, HbA1c, renal function, type 2 diabetes

## Abstract

**Aims** Sodium–glucose co-transporter 2 (SGLT2) reabsorbs glucose and sodium in the renal proximal tubule. Dapagliflozin, an SGLT2 inhibitor, targets hyperglycaemia in type 2 diabetes by increasing renal glucose excretion. To investigate whether the parallel occurring sodium loss would have diuretic-like physiologic effects, we compared dapagliflozin and hydrochlorothiazide (HCTZ) effects on 24-h blood pressure (BP), body weight, plasma volume and glomerular filtration rate (GFR).

**Methods** In this randomized, placebo-controlled, double-blind trial, 75 subjects with type 2 diabetes were assigned placebo, dapagliflozin 10 mg/day, or HCTZ 25 mg/day. Changes from baseline BP, body weight, plasma volume and GFR were assessed after 12 weeks of treatment.

**Results** Subjects’ mean age was 56 years, type 2 diabetes mellitus (T2DM) duration 6.3 years, and haemoglobin A1c (HbA1c) 7.5%. Treatment with placebo, dapagliflozin or HCTZ resulted in changes from baseline in 24-h ambulatory mean systolic blood pressure (SBP) of −0.9 (95%CI −4.2, +2.4), −3.3 (95%CI −6.8, +0.2), and −6.6 (95%CI −9.9, −3.2) mmHg, respectively at week 12, adjusted for baseline SBP. Body weight decreased with dapagliflozin and HCTZ. In a sub-study plasma volume appeared to decrease with dapagliflozin but did not change with placebo or HCTZ treatment. Dapagliflozin induced a greater reduction in GFR (−10.8%; 95%CI −14.6, −6.7) relative to placebo (−2.9%; 95% CI −6.9, +1.2) or HCTZ (−3.4%; 95%CI −7.3, +0.6).

**Conclusions** Dapagliflozin-induced SGLT2 inhibition for 12 weeks is associated with reductions in 24-h BP, body weight, GFR and possibly plasma volume. Cumulatively, these effects suggest that dapagliflozin may have a diuretic-like capacity to lower BP in addition to beneficial effects on glycaemic control.

## Introduction

Many patients with diabetes present with hyperglycaemia, hypertension, hypercholesterolemia and excess weight, which are associated with micro- and macrovascular complications. Therapies to alleviate the burden of these complications have traditionally focused on reducing glycaemia and optimizing blood pressure (BP) and cholesterol concentration. However, despite the various available treatment options to improve glycaemic control, many patients do not reach treatment targets. In addition, sulphonylurea derivatives and glitinides, which increase secretion of insulin, are associated with clinically important side effects such as weight gain and hypoglycaemia 1,2. Novel treatment strategies are therefore required that aid in achieving therapeutic targets without inducing weight gain or hypoglycaemia.

Emerging insight in the role of the sodium–glucose co-transporter 2 (SGLT2) in glucose reabsorption in the kidney has led to the development of selective orally available sodium–glucose transport inhibitors. These drugs are designed to inhibit SGLT2 located mainly in the S1-segment of the proximal tubule 4. This inhibition augments urinary glucose excretion, which has proved successful in reducing plasma glucose and haemoglobin A1c (HbA1c) 5,6. Inhibiting SGLT2, however, also leads to reduced sodium reabsorption in the proximal tubule; this can enhance sodium excretion. In previous studies 8,9 dapagliflozin administration has been associated with decreases in body weight and BP. Furthermore, dose-related increases in haematocrit and decreases in creatinine clearance were seen in dapagliflozin-treated patients 8. These observations suggest that a natriuretic effect of dapagliflozin may be associated with a ‘diuretic-like’ antihypertensive action.

The aim of this study was to assess whether dapagliflozin has diuretic-like effects along with its glucose-lowering effect. To this end, we determined the effects of dapagliflozin, hydrochlorothiazide and placebo on BP, body weight, plasma volume and renal function using dedicated measurement techniques.

## Methods

This was a multicenter, randomized, double-blind, three-arm, parallel-group, placebo- and active-controlled study conducted from October 2009 to July 2010 in Canada, The Netherlands and the USA. It is registered with http://www.clinicaltrials.gov (NCT00976495).

### Patients

Eligible patients were men and women with type 2 diabetes aged between 18 and 70 years, who had inadequate glycaemic control, defined as HbA1c ≥6.6% and ≤9.5%, and who were receiving a stable dose of metformin and/or a sulfonylurea derivative for at least 4 weeks prior to study entry. Subjects had to have an enrollment C-peptide ≥ 0.27 nmol/l, an estimated glomerular filtration rate (GFR) >60 ml/min/1.73 m^2^ and <150 ml/min/1.73 m^2^, urine albumin : creatinine ratio <300 mg/g, body mass index ≤45.0 kg/m^2^, and inadequate BP control, defined as systolic blood pressure (SBP) ≥130 and <165 mmHg, and/or diastolic BP ≥80 and <105 mmHg. Subjects with type 1 diabetes, those with cardiovascular disease within 6 months of study entry, and pregnant women were excluded from study participation. Subjects with a history of adverse reaction to radiocontrast dye, or allergy to or contraindication for thiazide diuretics were excluded as well. All subjects gave written informed consent prior to enrollment in the study. The study was approved by the appropriate local research ethics committee and was performed in accordance with the Declaration of Helsinki of the World Medical Association.

### Design

The study consisted of a 14-day qualification period, a 7-day single-blind lead-in period, and a double-blind treatment period of 12 weeks. A qualification assessment was performed in all patients that included a complete medical history, safety, laboratory measurements and pregnancy test for women of childbearing potential. Subjects maintained their stable dose of metformin and/or sulphonylurea derivatives. Subjects who were receiving a stable dose of angiotensin-converting enzyme inhibitors (ACEIs) or angiotensin receptor blockers (ARBs) continued these medications. Subjects received dietary counseling during the lead-in period and throughout the study. Twenty-four-hour ambulatory BP monitoring was initiated at the start of the lead-in period. Those patients who met the inclusion criteria and adhered to study medication were randomly assigned to dapagliflozin 10 mg/day, hydrochlorothiazide 25 mg/day, or matched placebo in a 1 : 1 : 1 ratio. Hydrochlorothiazide was chosen as active comparator as it is the most commonly used thiazide diuretic. Study treatments were allocated using a central, computer-based randomization procedure. Blinding of patients and investigators to study treatment was achieved using a double-dummy technique. Randomization was stratified globally by prior antihypertensive drug use (ACEI or ARB, or no antihypertensive drug) as well as participation in the red cell mass/plasma volume substudy. The doses of metformin and sulphonylurea derivatives as well as ACEI or ARB, if applicable, were maintained stable for the duration of the double-blind treatment period. Patients attended the clinic 1, 2, 4, 8 and 12 weeks after randomization. At each follow-up visit, efficacy and safety parameters were measured.

### Measurements

BP was measured by 24-h ambulatory BP monitoring (Spacelabs 90207 BP monitor). GFR was determined by plasma disappearance of iohexol. After a 5-ml bolus iohexol infusion (300 mg iodine/ml) blood samples were taken until 4 h after the iohexol infusion. Plasma iohexol concentrations were measured at a central laboratory (Karolinska Institute, Uppsala, Sweden) by high-performance liquid chromatography. Plasma iohexol was calculated according to a one-compartment model to obtain GFR (after correction according the Brochner–Mortensen formula and body surface area) 10. In subjects receiving metformin, the dose of metformin was withheld on the visit day of the GFR procedure until at least 48 h after the GFR procedure according the metformin label which directs that metformin should be temporarily discontinued prior to any intravascular radiocontrast study. Estimated GFR (eGFR) was calculated with the MDRD-formula. Plasma volume and red cell mass were simultaneously estimated using radioisotope techniques with ^51^Cr-labelled erythrocytes and ^125^I-labelled human serum albumin. All plasma volume and red cell mass measurements were performed at local sites. Safety and tolerability were assessed by collating data on adverse events using the Medical Dictionary for Regulatory Activities (MedDRA version 12.1).

### Statistical Analysis

The study was exploratory in nature and designed as a mechanism-of-action study to assess the effects of dapagliflozin on renal function, BP and circulating plasma volume. The objectives of the data analyses are primarily descriptive, and the planned total sample size of 75 subjects was determined based on administrative and logistical grounds. Confidence intervals are provided herein to facilitate the interpretation of the results, but the sample size was not intended to be sufficient to reach definitive statistical conclusions. Therefore, no statistical comparisons between treatment arms were made.

The analyses evaluated the change from baseline at week 12 in 24-h ambulatory BP, day-time and night-time BP, GFR, plasma volume and red cell mass. To account for differences in baseline values, baseline adjusted mean change in 24-h, day-time and night-time BP was analysed using an analysis of covariance (ancova) model with treatment group as an effect and baseline BP as a covariate. Baseline adjusted percentage mean change in GFR was analysed using an ancova model with treatment group and prior drug use (ACEI or ARB therapy or no antihypertensive drug use) as an effect and baseline GFR as a covariate. For the purpose of analysis, GFR values were transformed to logarithms and the changes from baseline were expressed as geometric mean percent changes from baseline. If no week 12 measurement was available, the last available postbaseline measurement was used for analysis of 24-h, day-time and night-time BP, and the last available postbaseline measurement after week 4 was used for analysis of GFR, plasma volume and red cell mass. Median changes in 24-h ambulatory BP, plasma volume and red cell mass are reported in the substudy because of the small sample size of the substudy and the fact that mean changes can be driven by a few extreme values. Analyses were conducted with SAS version 8.2.

## Results

Of the 154 patients enrolled, 75 entered the double-blind treatment period (fig. S1, Supporting Information). The majority of the subjects were male, white and under 65 years of age. Subjects in the placebo group were slightly older than those in the other two treatment groups (Table1). Mean baseline SBP were 127 (SD 12), 131 (12) and 124 (11) mmHg for the placebo, dapagliflozin 10 mg and hydrochlorothiazide 25 mg groups, respectively.

**Table 1:** Baseline characteristics of the study population

	Placebo (n = 25)	Dapagliflozin (n = 24)	Hydrochlorothiazide (n = 26)
Age, years	58.0 (9.5)	53.7 (9.4)	54.8 (9.9)
Sex, male; n, (%)	18 (72.0)	16 (66.7)	15 (57.7)
Duration of diabetes, years	6.5 (5.0)	6.5 (4.4)	6.0 (5.0)
HbA1c, %	7.5 (1.0)	7.7 (0.6)	7.4 (0.9)
Fasting plasma glucose, mmol/l	8.1 (2.4)	8.8 (2.0)	8.8 (2.5)
24-h Systolic blood pressure, mmHg	127 (12)	131 (12)	124 (11)
24-h Diastolic blood pressure, mmHg	74 (7)	77 (7)	71 (8)
Day-time systolic blood pressure, mmHg	131 (12)	138 (12)	130 (11)
Night-time systolic blood pressure, mmHg	117 (15)	120 (13)	114 (13)
Office systolic blood pressure, mmHg	132 (12)	141 (16)	133 (14)
Office diastolic blood pressure, mmHg	82 (8)	82 (9)	78 (8)
Glomerular filtration rate, ml/min/1.73 m^2^	100.6 (17.7)	100.6 (14.3)	101.9 (17.6)
Serum creatinine, µmol/l	76.9 (15.0)	76.9 (15.0)	72.5 (15.0)
Body weight, kg	96.2 (19.5)	93.2 (18.0)	96.2 (20.2)
Hematocrit, %	41.3 (3.5)	41.2 (2.7)	40.7 (5.1)
Hemoglobin, g/dl	14.0 (1.2)	13.9 (1.0)	13.9 (1.7)
Serum Erythropoietin, mU/ml	15.5 (10.5 − 19.5)	15.0 (10.0 − 21.5)	14.0 (10.0 − 19.0)
Metformin use, n (%)	25 (100)	22 (91.7)	25 (96.2)
Sulfonylurea use, n (%)	11 (44.0)	11 (45.8)	14 (53.8)
ACEI/ARB use, n (%)	17 (68.0)	16 (66.7)	18 (69.2)
Substudy population	n = 10	n = 9	n = 11
Age, years	63.4 (3.2)	52.7 (10.2)	53.8 (10.7)
Gender, male	9 (90.0)	7 (77.8)	4 (36.4)
HbA1c, %	7.4 (0.78)	7.8 (0.62)	7.2 (0.85)
24-h Systolic blood pressure, mmHg	131 (11)	133 (13)	122 (12)
24-h Diastolic blood pressure, mmHg	74 (6)	76 (8)	69 (9)
Body weight, kg	99.5 (19.0)	99.4 (23.8)	98.4 (22.7)
Plasma volume, ml	3001 (585)	2909 (411)	2889 (1027)
Red cell mass, ml	2494 (541)	2299 (783)	2071 (500)

Data are given as mean and standard deviation unless otherwise indicated. Because of an extreme outlier, the median baseline serum erythropoietin concentration (25th and 75th percentile) is reported.

### Changes in Blood Pressure, Body Weight and Plasma Volume

**Figure 1:**
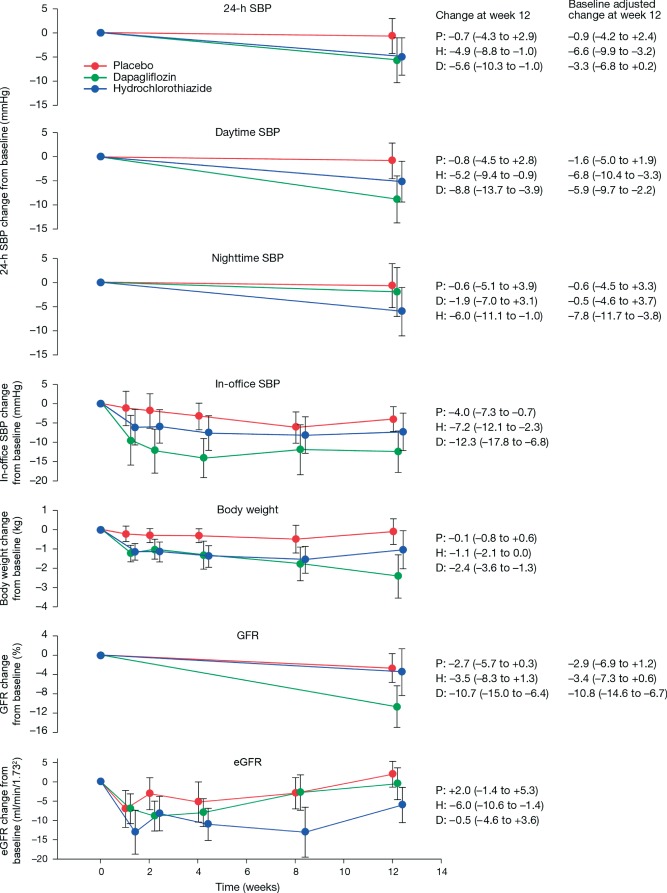
Mean change in 24-h, day-time and night-time ambulatory systolic blood pressure, body weight, in-office systolic blood pressure and glomerular filtration rate over the course of the study in placebo, dapagliflozin and hydrochlorothiazide groups. Data are reported as mean (± 95% CI). P, placebo; D,  dapagliflozin; H, hydrochlorothiazide.

After 12 weeks, treatment with dapagliflozin and hydrochlorothiazide resulted in 24-h ambulatory SBP changes from baseline of −5.6 mmHg (95% CI −10.3 to −1.0) and −4.9 mmHg (−8.8 to −1.0). In the placebo group, a small decrease in ambulatory SBP was observed at week 12 of −0.7 mmHg (−4.3 to +2.9). After adjustment for baseline 24-h SBP, treatment with placebo, dapagliflozin and hydrochlorothiazide resulted in changes of −0.9 mmHg (95% CI −4.2 to +2.4), −3.3 mmHg (−6.8 to +0.2) and −6.6 mmHg (−9.9 to −3.2), respectively. Different results were obtained for day-time and night-time ambulatory SBP: the mean change from baseline in day-time ambulatory SBP at week 12 in the dapagliflozin and hydrochlorothiazide groups was greater than the change in the placebo group, whereas a decrease in night-time BP only occurred in the hydrochlorothiazide group and not in the dapagliflozin group (figure 1). Subgroup analysis of concurrent antihypertensive drug use (those on no antihypertensive therapy vs. those with background ACEI or ARB therapy) showed similar decreases in SBP in the dapagliflozin and hydrochlorothiazide groups regardless of prior antihypertensive drug use. Mean reductions from baseline in office systolic/diastolic BP at week 12 were generally greater in the dapagliflozin group (−12.3/−5.1 mmHg) than in the hydrochlorothiazide or (−7.2/−1.6 mmHg) or placebo groups (−4.0/−3.1 mmHg) (figure 1).

**Figure 2:**
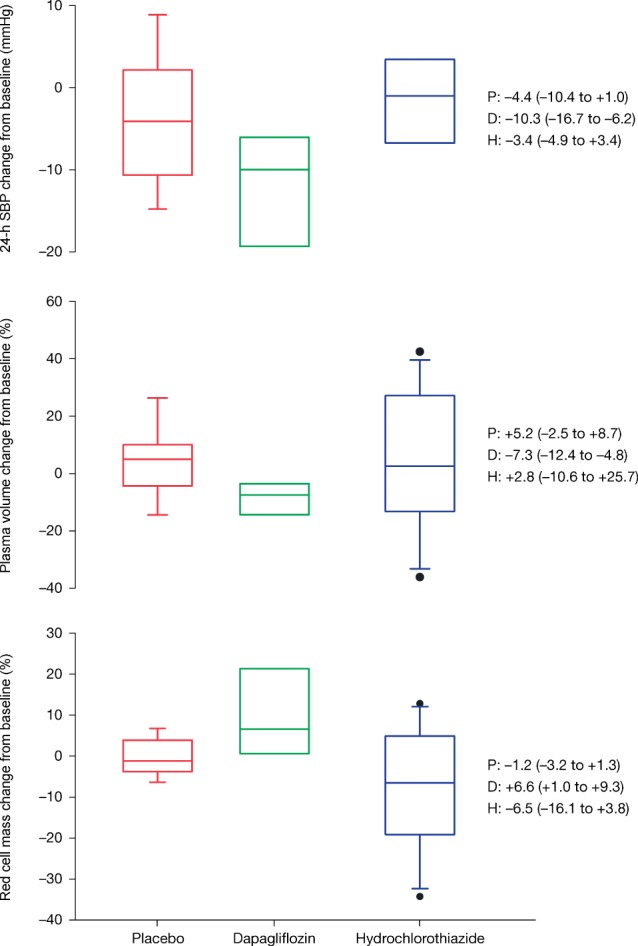
Median change in 24-h systolic blood pressure and median percentage change in plasma volume and red cell mass in substudy participating subjects in the placebo, dapagliflozin and hydrochlorothiazide groups. Because of the small sample size, data are reported as median (25th and 75th percentile) in order to exclude the influence of few extreme values that drive mean changes. In two patients plasma volume change could not be measured because an end of study measurement was not available. As a result, the sample size was too small to show 2.5–97.5% percentile. P, placebo; D, dapagliflozin; H, hydrochlorothiazide.

To determine whether the BP-lowering effects of dapagliflozin reflect a reduction in extracellular volume, body weight and levels of volume regulating hormones were measured. In addition, plasma volume was measured in a substudy of 30 subjects. Body weight did not appreciably change over time in the placebo group (figure 1). In the dapagliflozin and hydrochlorothiazide groups, reductions from baseline in body weight occurred in the first week that plateaued in the hydrochlorothiazide group. In the dapagliflozin group, the initial 1-week reduction in body weight was followed by a more gradual reduction that persisted throughout follow-up (figure 1). In the substudy, the reduction in 24-h SBP was smaller in the hydrochlorothiazide group than in the other two treatment groups (figure 2). The median (interquartile range) percentage change from baseline in plasma volume were +5.2% (−2.5 to +8.7), −7.3% (−12.4 to −4.8) and +2.8% (−10.6 to +25.7) for the placebo, dapagliflozin and hydrochlorothiazide groups, respectively, compatible with a reduction in circulating volume in the dapagliflozin group (figure 2). Consistent with the observed changes in body weight and plasma volume was the 12-week increase in plasma renin activity and serum aldosterone in the dapagliflozin group (Table2). NT-proBNP concentration increased slightly in the dapagliflozin group and did not change in the placebo and hydrochlorothiazide groups (Table2).

**Table 2:** Week 12 changes from baseline in extracellular volume related parameters and metabolic parameters in placebo, dapagliflozin and hydrochlorothiazide treatment arms

	Placebo (n = 25)	Dapagliflozin 10 mg (n = 24)	Hydrochlorothiazide 25 mg (n = 26)
*Extracellular volume-related parameters*			
NT-ProBNP, ng/l			
Baseline, median (25th–75th percentile)	33.0 (20–42)	22.0 (12–52)	20.0 (13–45)
Change at week 12, median (IQR)	3.0 (30.0)	19.0 (32.0)	−3.0 (21.0)
Plasma renin activity, × 10^−2^ pmol/l/h			
Baseline, median (25th–75th percentile)	2.84 (0.71–17.8)	2.13 (1.19–4.03)	2.37 (1.42–9.48)
Change at week 12, median (IQR)	−0.23 (8.53)	0.71 (12.1)	3.08 (9.01)
Serum aldosterone, pmol/l			
Baseline, median (25th–75th percentile)	277.4 (221.9–360.6)	374.5 (249.7–499.3)	332.9 (249.7–457.7)
Change at week 12, median (IQR)	−55.5 (221.9)	55.5 (152.6)	55.5 (166.4)
*Metabolic parameters*			
HbA1c, %			
Baseline, mean (SD)	7.5 (1.0)	7.7 (0.6)	7.4 (0.9)
Change at week 12, mean (95% CI)	−0.4 (−0.7 to −0.1)	−0.7 (−1.1 to −0.4)	0.1 (−0.2 to +0.5)
Fasting plasma glucose, mmol/l			
Baseline, mean (SD)	8.06 (2.39)	8.79 (2.00)	8.74 (2.52)
Change at week 12, mean (95% CI)	0.43 (−0.24 to +1.09)	−1.33 (−2.12 to −0.54)	0.19 (−0.67 to +1.05)

Extracellular volume-related parameters are reported as median [interquartile range (IQR)] and metabolic parameters as mean (95% CI).

### Change in Renal Function

By the end of the 12-week treatment period, decreases in mean GFR were observed in all treatment groups (figure 1), with a greater baseline adjusted percentage change in mean GFR in the dapagliflozin group (−10.8%; 95% CI −14.6 to −6.7) relative to placebo (−2.9%; 95% CI −6.9 to +1.2) or hydrochlorothiazide (−3.4%; 95% CI −7.3 to +0.6). Subgroup analysis by concurrent antihypertensive medication use produced similar results. eGFR decreased from baseline in the dapagliflozin and hydrochlorothiazide group up to week 2. From week 2, it slightly increased in the dapagliflozin group and remained relatively stable in the hydrochlorothiazide group (figure 1).

### Haematopoiesis

**Figure 3:**
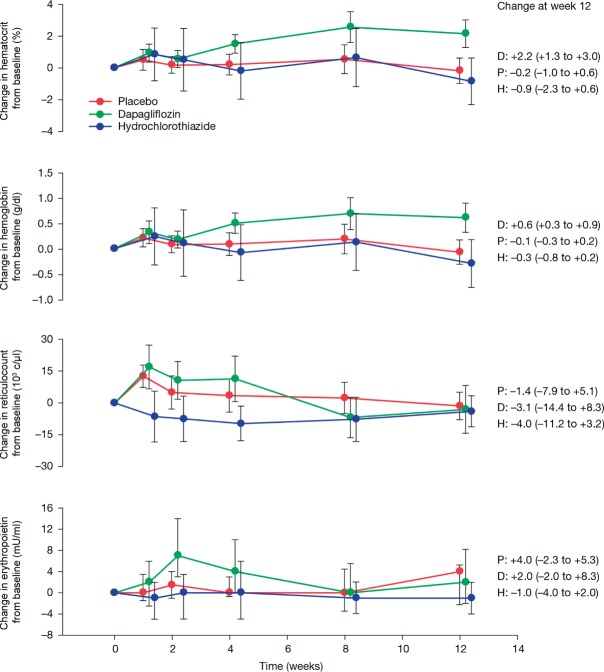
Mean change in haematocrit, haemoglobin, reticulocyte count and erythropoietin over the course of the study in placebo dapagliflozin, and hydrochlorothiazide groups. Data are reported as mean (±95% CI). Because of an extreme outlier, the median concentrations (25th and 75th percentile) in erythropoietin over time are reported in the figure. Excluding the outlier and calculating the mean value provided a similar picture (data not shown). P, placebo; D, dapagliflozin; H, hydrochlorothiazide.

To add to the understanding of small increases in haematocrit that were observed in previous studies with dapagliflozin, haematocrit, haemoglobin, serum erythropoietin concentrations, reticulocytes, and, in a substudy, red cell mass were measured. At week 12, haematocrit increased by 2.2 (95% CI 1.3 to 3.0) in the dapagliflozin group, compared with changes of −0.2 (−1.0 to +0.6) and −0.9 (−2.3 to +0.6) in the placebo and hydrochlorothiazide group (figure 3). Parallel changes in mean haemoglobin levels were also observed (figure 3). Mean reticulocyte count and serum erythropoietin transiently increased from baseline in the dapagliflozin group up to week 4, followed by a gradual decline until week 12. The median percent changes (interquartile range) from baseline in red cell mass were −1.2% (−3.2 to +1.3), +6.6% (1.0 to 9.3) and −6.5% (−16.1 to +3.8) for the placebo, dapagliflozin and hydrochlorothiazide groups, respectively (figure 2), although the small sample size hampers precise quantification.

### Changes in Metabolic Parameters

Improvements in HbA1c and fasting plasma glucose were observed at week 12 relative to baseline in subjects treated with dapagliflozin (Table2). As expected, HbA1c and plasma glucose fell with dapagliflozin and slightly increased with hydrochlorothiazide. A post hoc regression analysis showed that 12-week changes in fasting plasma glucose did not correlate with changes in 24-h SBP in any of the three treatment groups: placebo (*R*^2^ = 0.016; p = 0.55), dapagliflozin (*R*^2^ = 0.003; p = 0.83), hydrochlorothiazide (*R*^2^ = 0.019; p = 0.55).

### Safety

The proportion of subjects with at least one adverse event was higher in the active treatment groups than in the placebo group. The proportion of subjects for whom adverse events were considered related to study medication was higher in the active treatment groups than in the placebo groups (9 of 24, 37.5% for dapagliflozin, 9 of 26, 34.6% for hydrochlorothiazide, vs. 4 of 25, 16.0% for placebo). One subject in the dapagliflozin group and two subjects in the hydrochlorothiazide group had events suggestive of urinary tract infection during the study. These events were considered related to treatment with the study medication by the investigator and were mild in intensity. Events suggestive of genital infection were reported for two subjects in the dapagliflozin group and in no subjects in the other two treatment groups. Adverse events of hypoglycaemia were reported by three subjects in the placebo group, one subject in the dapagliflozin group, and one subject in the hydrochlorothiazide group. There was one report of dizziness and syncope in the dapagliflozin arm which was considered related to study medication according the investigator’s judgment. No adverse events led to discontinuation of study medication in any treatment group. Three serious adverse events (right leg cellulitis, surgery for lumbar disc strain and pleuritic chest pain) occurred in the dapagliflozin group and in no subjects in the other two treatment groups. None of these serious adverse events were considered related to study medication according the investigator’s judgment. No deaths occurred during the study.

## Discussion

This exploratory study employed dedicated measures to assess the effects of dapagliflozin, hydrochlorothiazide and placebo on BP, plasma volume and renal function. Treatment with dapagliflozin and hydrochlorothiazide reduced mean 24-h SBP and in-office BP from baseline. In addition, body weight fell and, in a substudy, dapagliflozin appeared to be associated with a reduction in plasma volume. After 12 weeks treatment an approximately 10% reduction in GFR was observed with dapagliflozin. Taken together, these results imply that the reduction in BP with dapagliflozin therapy is associated with a ‘natriuretic/diuretic-like’ effect.

Previous studies have shown greater reductions of office SBP with dapagliflozin treatment than placebo treatment 7–8. In this study, we employed ambulatory BP monitoring to allow for more precise estimation of the magnitude and the time-course of blood-pressure effects, in addition to office BP measurements. A greater decrease from baseline in mean systolic 24-h ambulatory BP was observed with dapagliflozin than hydrochlorothiazide. However, after adjusting for the differences in baseline SBP, the reduction was greater with hydrochlorothiazide. Day-time reduction in SBP was similar with dapagliflozin and hydrochlorothiazide. There was no reduction in night-time BP in dapagliflozin- and placebo-treated subjects, while persistent reductions were observed in hydrochlorothiazide-treated subjects. This observed difference is unlikely to reflect pharmacokinetic-pharmacodynamic considerations given the steady 24-h glucosuria induced by dapagliflozin 5–6. Whether this observed difference can be explained by diminished dapagliflozin BP effect related to the lower BPs experienced at night or by a diminished volume-driven BP effect in the recumbent position 11 cannot be ascertained from this study and requires further investigation. Mean reductions from baseline in seated in-office BP were somewhat greater for the dapagliflozin group than for the hydrochlorothiazide group. Seated in-office BP measurements were conducted during day-time which may explain the somewhat larger reduction with dapagliflozin compared to hydrochlorothiazide. The reduction in both ambulatory BP and in-office pressures indicate the antihypertensive potential of dapagliflozin. This effect may be beneficial for the majority of patients with type 2 diabetes who are often diagnosed with hypertension, although caution is warranted for a too low BP in certain populations such as the elderly or those with comorbidities.

The reduction in BP seen with dapagliflozin may well reflect a diuretic effect as a result of enhanced sodium excretion during SGLT2 blockade. A previous healthy volunteer study has shown dose-dependent effects of dapagliflozin on cumulative sodium excretion 6. The initial reduction in body weight in the dapagliflozin and hydrochlorothiazide arm provides further support for the diuretic effects of both agents. Dapagliflozin has been shown to induce a reduction in body weight in patients with type 2 diabetes. This weight loss appears to be biphasic: an initial steep fall followed by a more gradual continuous reduction. The increased excretion of glucose and associated calorie loss appear to be mainly responsible for the reduction in body weight, as substantiated in a previous study 9, although the initial reduction in body weight can be attributed to fluid loss.

Plasma volume measurements were utilized to further characterize the diuretic effects of dapagliflozin and to compare those with a registered diuretic. The results suggest a 7% reduction in plasma volume with dapagliflozin treatment indicating a diuretic effect possibly owing to enhanced sodium excretion or osmotic diuresis as a result of increased glucose excretion. Dapagliflozin treatment was associated with increases in plasma renin activity and serum aldosterone concentration, as can be expected with diuretic-induced reductions in plasma volume. A slight increase in NT-pro-BNP was seen with dapagliflozin which is discordant with a reduction in circulating volume. A possible explanation may be the transient rise in erythropoietin which has been shown to enhance brain natriuretic peptide secretion 12; however, the small sample size and large response variability warrant cautious interpretation. It should be noted that a lack of a significant reduction in plasma volume with hydrochlorothiazide has been observed in previous long-term hemodynamic studies as well, irrespective of the BP response 13. The absence of such a reduction in plasma volume with hydrochlorothiazide can be explained by either tolerance to the effects of prolonged hydrochlorothiazide treatment or the establishment of compensatory mechanisms to restore fluid balance 14.

A decrease in plasma volume, with resultant haemoconcentration, could contribute to an increase in haematocrit as observed in the present and previous studies 8. However, an alternative explanation for the rise in haematocrit may be an effect of dapagliflozin on red cell mass, as suggested by the transient increases in reticulocyte count and serum erythropoietin concentrations.

Dapagliflozin and Hydrochlorothiazide caused reductions in iohexol measured GFR. The reduction in iohexol measured GFR with dapagliflozin treatment could be the consequence of increased sodium delivery at the macula densa. As a result, tubulo-glomerular feedback causes a fall in renal plasma flow, and reduction in intraglomerular pressure and GFR 15,16. Alternatively, reductions in systemic BP or plasma volume (secondary to the diuretic effects of dapagliflozin) coupled with imperfect renal autoregulation could lead to reductions in intraglomerular pressure, as can be seen in diabetic patients 18.

Renal function was measured at baseline and week 12 which precluded the possibility to assess the time-course of the effect of dapagliflozin on GFR. eGFR was measured throughout the study. The difference in absolute values between measured GFR and eGFR illustrate the limitations of the latter in estimating GFR in patients with normal or near-normal renal function 19. However, eGFR can be used to assess the changes within an individual over time. The observed changes in eGFR over time suggest that dapagliflozin and hydrochlorothiazide caused an acute fall in renal function which was maximally present after 2 weeks therapy and did not further decline during the remainder of follow-up. This observation is in line with a meta-analysis on the effect of dapagliflozin on renal function that included 12 placebo controlled randomized studies and involved more than 4000 patients with preserved renal function. In that analysis dapagliflozin caused a fall in eGFR at week 1 that slowly returned to baseline by week 24 and maintained at baseline for 2 years 20. Another study in subjects with type 2 diabetes and renal impairment showed that dapagliflozin administration caused a rapid decline in eGFR, within 2 weeks, followed by a period of minimal change in eGFR up to 52 weeks therapy 21. The pattern of acute eGFR decline followed by stabilization suggests that the acute eGFR decline reflects a (reversible) haemodynamic change rather than worsening of structural renal function 22. However, GFR was not measured after treatment cessation so that we are unable to ascertain the reversibility of the initial fall in GFR and can only speculate about a potential hemodynamic induced reversible GFR effect.

Changes in metabolic parameters such as fasting plasma glucose, HbA1c and body weight were not the primary focus of this study. However, as expected, treatment with dapagliflozin caused reductions in fasting plasma glucose as well as HbA1c.

The study is limited by the fact that the small sample size does not allow definitive conclusions; it merely is hypothesis generating. In addition, consecutive 24-h urines were not collected and dietary intake of sodium was not recorded so that natriuretic effects of dapagliflozin could not be directly quantified, nor compared with hydrochlorothiazide. Third, GFR was not measured post-treatment to assess the reversibility of the initial fall in GFR with dapagliflozin or hydrochlorothiazide. Finally, the sub-study population was in general representative of the overall population although the proportion of male subjects was slightly higher in the placebo and dapagliflozin group and somewhat lower in the hydrochlorothiazide group.

In conclusion, along with glycaemic control, SGLT2 inhibition with dapagliflozin for 12 weeks was associated with reductions in BP, body weight, GFR and possibly plasma volume. These findings suggest that the BP–lowering effect of dapagliflozin could be driven by a decrease in circulating volume owing to diuretic/natriuretic properties of the drug. Further mechanistic studies are warranted.

## Supporting Information

Figure S1 Trial profile.

## References

[b1] Feinglos M, Dailey G, Cefalu W (2005). Effect on glycemic control of the addition of 2.5 mg glipizide GITS to metformin in patients with T2DM. Diabetes Res Clin Pract.

[b2] Hamann A, Garcia-Puig J, Paul G, Donaldson J, Stewart M (2008). Comparison of fixed-dose rosiglitazone/metformin combination therapy with sulphonylurea plus metformin in overweight individuals with type 2 diabetes inadequately controlled on metformin alone. Exp Clin Endocrinol Diabetes.

[b3] Phung OJ, Scholle JM, Talwar M, Coleman CI (2010). Effect of noninsulin antidiabetic drugs added to metformin therapy on glycemic control, weight gain, and hypoglycemia in type 2 diabetes. JAMA.

[b4] Bakris GL, Fonseca VA, Sharma K, Wright EM (2009). Renal sodium-glucose transport: role in diabetes mellitus and potential clinical implications. Kidney Int.

[b5] Komoroski B, Vachharajani N, Boulton D (2009). Dapagliflozin, a novel SGLT2 inhibitor, induces dose-dependent glucosuria in healthy subjects. Clin Pharmacol Ther.

[b6] Komoroski B, Vachharajani N, Feng Y, Li L, Kornhauser D, Pfister M (2009). Dapagliflozin, a novel, selective SGLT2 inhibitor, improved glycemic control over 2 weeks in patients with type 2 diabetes mellitus. Clin Pharmacol Ther.

[b7] List JF, Woo V, Morales E, Tang W, Fiedorek FT (2009). Sodium-glucose cotransport inhibition with dapagliflozin in type 2 diabetes. Diabetes Care.

[b8] Bailey CJ, Gross JL, Pieters A, Bastien A, List JF (2010). Effect of dapagliflozin in patients with type 2 diabetes who have inadequate glycaemic control with metformin: a randomised, double-blind, placebo-controlled trial. Lancet.

[b9] Bolinder J, Ljunggren O, Kullberg J (2012). Effects of dapagliflozin on body weight, total fat mass, and regional adipose tissue distribution in patients with type 2 diabetes mellitus with inadequate glycemic control on metformin. J Clin Endocrinol Metab.

[b10] Brochner-Mortensen J (1972). A simple method for the determination of glomerular filtration rate. Scand J Clin Lab Invest.

[b11] Ogi M, Kojima S, Kuramochi M (1998). Effect of postural change on urine volume and urinary sodium excretion in diabetic nephropathy. Am J Kidney Dis.

[b12] Piuhola J, Kerkela R, Keenan JI, Hampton MB, Richards AM, Pemberton CJ (2008). Direct cardiac actions of erythropoietin (EPO): effects on cardiac contractility, BNP secretion and ischaemia/reperfusion injury. Clin Sci (Lond).

[b13] Gifford RW, Mattox VR, Orvis AL, Sones DA, Rosevear JW (1961). Effect of thiazide diuretics on plasma volume, body electrolytes, and excretion of aldosterone in hypertension. Circulation.

[b14] Conway J, Lauwers P (1960). Hemodynamic and hypotensive effects of long-term therapy with chlorothiazide. Circulation.

[b15] Vallon V, Blantz RC, Thomson S (2003). Glomerular hyperfiltration and the salt paradox in early [corrected] type 1 diabetes mellitus: a tubulo-centric view. J Am Soc Nephrol.

[b16] Vallon V, Richter K, Blantz RC, Thomson S, Osswald H (1999). Glomerular hyperfiltration in experimental diabetes mellitus: potential role of tubular reabsorption. J Am Soc Nephrol.

[b17] Pollock CA, Lawrence JR, Field MJ (1991). Tubular sodium handling and tubuloglomerular feedback in experimental diabetes mellitus. Am J Physiol.

[b18] Christensen PK, Hansen HP, Parving HH Impaired autoregulation of GFR in hypertensive non-insulin dependent diabetic patients. Kidney Int.

[b19] Gaspari F, Perico N, Matalone M (1997). Precision of plasma clearance of iohexol for estimation of GFR in patients with renal disease. J Am Soc Nephrol.

[b20] Ptaszynska A, Chalamandaris A-G, Sugg JE, Johnson KM, Parikh SJ, List J

[b21] Kohan DE, Fioretto P, List J, Tang W (2011). Efficacy and safety of dapagliflozin in patients with type 2 diabetes and moderate renal impairment [abstract]. J Am Soc Nephrol.

[b22] Lambers Heerspink HJ, De Zeeuw D (2010). Composite renal endpoints: was ACCOMPLISH accomplished?. Lancet.

