# Supplementary figures and images for: Dapagliflozin a glucose-regulating drug with diuretic properties in subjects with type 2 diabetes
Source: Diabetes Obes Metab. 2013 Jun 5;15(9):853–62. doi: 10.1111/dom.12127 (PMC3906841; doi:10.1111/dom.12127)

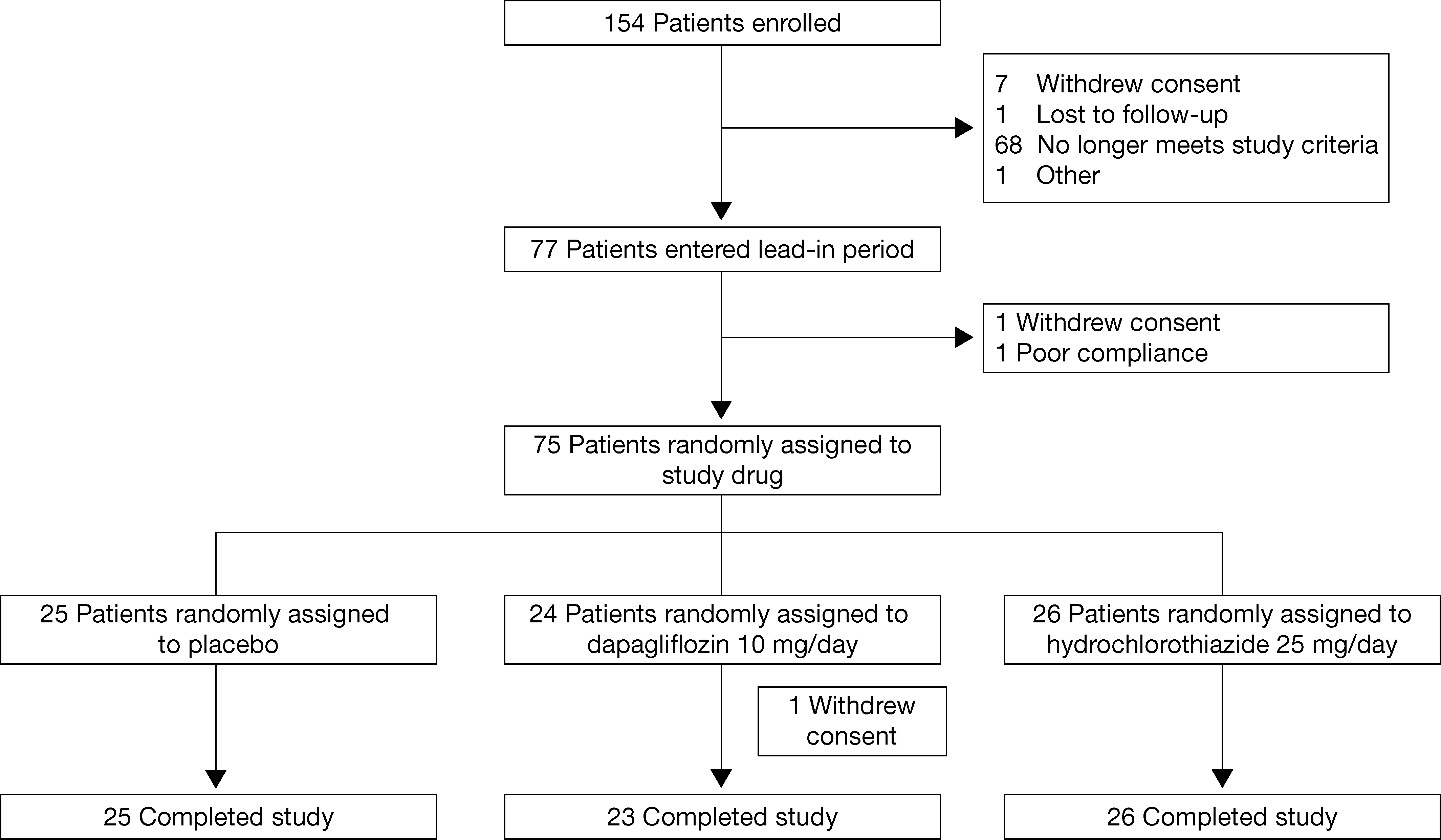

Supplement: Figure S1 — Trial profile. [file dom0015-0853-sd1.tif]
